# Basic Fibroblast Growth Factor Ameliorates Endothelial Dysfunction in Radiation-Induced Bladder Injury

**DOI:** 10.1155/2015/967680

**Published:** 2015-08-13

**Authors:** Shiwei Zhang, Xuefeng Qiu, Yanting Zhang, Kai Fu, Xiaozhi Zhao, Jinhui Wu, Yiqiao Hu, Weiming Zhu, Hongqian Guo

**Affiliations:** ^1^Department of Urology, Affiliated Drum Tower Hospital, School of Medicine, Nanjing University, Nanjing 210008, China; ^2^Institute of Urology, Nanjing University, Nanjing 210008, China; ^3^Department of Surgery, Jinling Hospital, School of Medicine, Nanjing University, Nanjing 210002, China; ^4^State Key Laboratory of Pharmaceutical Biotechnology, Nanjing University, Nanjing 210093, China

## Abstract

This study was designed to explore the effect of basic fibroblast growth factor (bFGF) on radiation-induced endothelial dysfunction and histological changes in the urinary bladder. bFGF was administrated to human umbilical vein cells (HUVEC) or urinary bladder immediately after radiation. Reduced expression of thrombomodulin (TM) was indicated in the HUVEC and urinary bladder after treatment with radiation. Decreased apoptosis was observed in HUVEC treated with bFGF. Administration of bFGF increased the expression of TM in HUVEC medium, as well as in the urinary bladder at the early and delayed phases of radiation-induced bladder injury (RIBI). At the early phase, injection of bFGF increased the thickness of urothelium and reduced inflammation within the urinary bladder. At the delayed phase, bFGF was effective in reducing fibrosis within the urinary bladder. Our results indicate that endothelial dysfunction is a prominent feature of RIBI. Administration of bFGF can ameliorate radiation-induced endothelial dysfunction in urinary bladder and preserve bladder histology at early and delayed phases of RIBI.

## 1. Introduction

Radiotherapy is a well-established therapy for treatment of more than half of all cancers. However, side effects due to radiation treatment are unavoidable, even with localized radiotherapy. Most patients treated with radiotherapy will develop acute complications and some chronic damage, such as fibrosis [1]. One of the organs that frequently is affected by pelvic radiotherapy is the urinary bladder because it is included in the radiation field of a wide variety of malignancies located in the rectum, prostate, uterus/cervix, and the bladder itself [1].

The early phase usually starts during treatment and resolves within a few weeks after the end of radiotherapy. This phase is characterized by inflammation within the bladder wall. Patients often exhibit increased urinary frequency, dysuria, and urgency [2]. After a symptom-free latent time that can extend up to 10 years or more, a progressive and irreversible delayed phase, characterized by fibrosis of the bladder and reduced bladder capacity, occurs [3]. The underlying pathogenetic mechanisms of RIBI, however, are not fully understood and few effective treatment strategies have been developed [4].

An increasing body of evidence describes that injury of endothelial cells plays a central role in radiation-induced injury in many normal tissues [5]. Radiation can induce endothelial dysfunction manifested as endothelial apoptosis, detachment, and increased endothelial permeability [6]. In the radiated urinary bladder, inflammation changes, such as intercellular adhesion molecule-1 [7] and cyclooxygenase-2 [8], vasodilatation [8], and barrier dysfunction [9] have been detected in blood vessels, suggesting the potential role of endothelial dysfunction in the pathogenesis of RIBI.

Recently, growth factors have been tested for their potential to reduce radiation-induced injuries. As an endothelial protector, basic fibroblast growth factor (bFGF) has been extensively studied for amelioration of radiation effects in various organs [10, 11]. It has been demonstrated that bFGF was effective in protecting endothelial cells against radiation-induced apoptosis in vitro and in vivo [12, 13]. Fibroblast growth factor-7 (FGF-7), a member of the fibroblast growth factor family, can modify radiation-induced early functional changes, as well as late effects in mouse urination [14]. However, the effect of bFGF on radiation-induced endothelial dysfunction and histological changes in the urinary bladder has not yet been evaluated.

Thrombomodulin (TM) is a transmembrane glycoprotein located on the luminal surface of endothelial cells in most healthy blood vessels. It has been indicated that TM plays a crucial role in mediating coagulation, inflammation, and fibrosis [6]. TM can also be an effective marker to reflect radiation-induced endothelial dysfunction [15, 16]. In the present study, we selected TM as a marker to evaluate radiation-induced endothelial dysfunction. We hypothesize that administration of bFGF is effective in ameliorating radiation-induced endothelial dysfunction, and subsequently, preserving bladder histology. To the best of our knowledge, this is the first study to explore the feasibility of using bFGF to ameliorate radiation-induced endothelial dysfunction in the urinary bladder.

## 2. Materials and Methods

### 2.1. Cells and Study Design

The human umbilical vein endothelial cell (HUVEC) line was purchased from Lonza Biologics Inc. (Portsmouth, NH, USA) and cultured in Dulbecco's modified Eagle's medium (DMEM, Invitrogen, Carlsbad, USA), supplemented with 10% fetal bovine serum (FBS, Invitrogen, Carlsbad, USA), 1% penicillin, and 1% streptomycin (Invitrogen, Carlsbad, USA). Culture incubator was set at 37°C with 5% CO_2_. HUVECs were divided into three groups: The control group received no radiation (C); the radiation group received radiation without any treatment (R); the radiation + bFGF group received radiation and treatment with basic fibroblast growth factor (bFGF, Invitrogen, 10 ng/mL, 24 hours before radiation and after radiation) (R+b).

### 2.2. Radiation Procedure for Cells

HUVECs were plated onto 96-well plates (5,000 cells/well). For the radiation assay, a single dosage of radiation (10 Gy) was delivered with a linear accelerator (Siemens, 6-MV X-ray, 2 Gy/min) according to the preliminary results (see Figure  S.1 in supplementary material available online at http://dx.doi.org/10.1155/2015/967680).

### 2.3. Detection of Cell Apoptosis

Determination of apoptosis in HUVEC was performed using the TUNEL staining kit (Roche, Basel, Switzerland) according to the instructions provided by the manufacturer. Briefly, the cells were washed with PBS and fixed with 4% paraformaldehyde three days after radiation procedure. After permeabilization with Triton X-100, the cells were incubated with the labeling solution. Nuclei were stained with 4′,6-diamidino-2-phenylindole (DAPI, Sigma-Aldrich).

To further quantify the apoptosis induced by radiation, Annexin-V/PI double staining assays were performed according to the manufacture's protocol (Biyuntian, Nantong, China). Briefly, after washing with PBS, HUVECs were resuspended in binding buffer (10 mM HEPES/NaOH, pH 7.4, 140 mM NaCl, 2.5 mM CaCl_2_) and then 10 *μ*L Annexin-V and 10 *μ*L PI were added. After incubation in darkness for 15 min, the cells were analyzed by using FACSCalibur (BD Bioscience, Sparks, MD, USA). Data was analyzed with the Cell Quest software (BD Biosciences).

### 2.4. Enzyme-Linked Immunosorbent Assay (ELISA)

HUVECs were plated onto 96-well plates (5,000 cells/well). After radiation, culture medium from each group was collected every day for the detection of TM. The concentration of TM in the HUVEC medium was assessed using a commercial available ELISA kit (Uscn Life Science Inc., Wuhan, China) following the instructions provided by the manufacturer.

Expressions of interleukin-1 *β* (IL-1*β*) and tumor necrosis factor-*α* (TNF-*α*) in the supernatant from bladder homogenate were also determined using ELISA kits (Uscn Life Science Inc). Protein content in the sample was determined by Coomassie blue assay and the results were corrected per microgram of protein.

### 2.5. Animals and Study Design

Fifty-two male Sprague-Dawley rats (12 weeks old) were housed in the animal facility at Nanjing University. All animal procedures were approved by the Institutional Animal Care and Use Committee at Nanjing University. Animals were randomly divided into three groups: control + PBS group received bladder injection of phosphate-buffered saline (PBS) (C, *n* = 16); radiation + PBS group received radiation over the bladder and injection of PBS (R, *n* = 18); radiation + bFGF group received radiation over the bladder and injection of bFGF (R+b, *n* = 18). PBS or bFGF was injected into urinary bladder immediately after the radiation procedure. To investigate the effect of bFGF at early phase [7, 8], half of the animals were euthanized 1 week after radiation and urinary bladder samples were collected for histological or molecular analysis. To investigate the effect of bFGF at delayed phase [7, 8], the remaining animals were euthanized and urinary samples were collected for histological or molecular analysis after functional test 12 weeks after radiation.

### 2.6. Radiation Procedure for Rats

Rats were anesthetized intraperitoneally with a mixture of ketamine (100 mg/kg) and midazolam (5 mg/kg). A lead shield with a 25 mm × 15 mm window was used to limit the radiation to the bladder. A single dosage of 20 Gy was delivered with a linear accelerator (Siemens, 6-MV X-ray, 2 Gy/min). The dosage of 20 Gy for the animal study was selected based on our preliminary results (Figure  S.3).

### 2.7. Bladder Injection of bFGF

All rats were anesthetized and underwent midline laparotomy to expose the urinary bladder. Rats underwent an injection of 1 mL PBS or 10 ng bFGF in 1 mL PBS into the muscular layer of bladder. After treatment, the incision was closed in 2 layers.

### 2.8. Histological Analysis

The middle part of the urinary bladder was fixed in 4% formaldehyde, dehydrated, and paraffin embedded. After dewaxing and rehydration, sections (5 *μ*m) were stained with hematoxylin and eosin (HE). For Masson's trichrome staining, the sections were stained using commercially available kits (Jiancheng, Nanjing, China) following the instructions provided by the manufacturer.

### 2.9. Immunofluorescent Analysis

After dewaxing and rehydration, the sections were incubated with 3% BSA/0.3% Triton X-100 for 30 min at room temperature. After draining this solution, the slides were incubated at room temperature with rabbit anti-myeloperoxidase (MPO, 1 : 100, Santa Cruz Biotechnologies, Santa Cruz, CA, USA), mouse anti-*α* smooth muscle actin (*α*-SMA) (1 : 1000, Sigma-Aldrich, St. Louis, MO, USA), rabbit anti-TM (1 : 100, Santa Cruz), or rabbit anti-CD31 (1 : 200, Santa Cruz) overnight. After rinsing with PBS, the sections were incubated with Alexa-488- or Alexa-594-conjugated secondary antibody (Life Science). After rinsing with PBS, the slides were incubated with DAPI.

### 2.10. Image Analysis and Quantification

Bladder sections were examined in a blinded manner. Data was averaged on at least 6 high power fields from each section and 5 sections from each bladder. Images were captured on a Nikon microscope with a Spot RT color digital camera, and digital histomorphometric analysis was performed using Image-Pro Plus 6.0 software (Media Cybernetics, Silver Spring, MD, USA).

### 2.11. Determination of Myeloperoxidase (MPO) Activity

Commercial available kit (Jiancheng Bioengineering Institute) was used to detect MPO, an indicator of neutrophil infiltration, in the bladder tissues according to the protocol provided by the manufacturer. MPO activity was defined as the quantity of enzyme degrading 1 *μ*mol of peroxide per minute at 37°C and was expressed in unit per milligram protein.

### 2.12. Metabolic Cage Evaluation

Rats were housed in the metabolic cage for 24 hours for the measurement of 24-hour urine output. According to a previously published protocol [17], the urine was collected in a container containing 5 mL of liquid paraffin to prevent evaporation.

### 2.13. Western Blot

Total protein was isolated from the bladder tissues. Briefly, freshly isolated bladder tissue was lysed in ice-cold extraction buffer (Beyotime Biotechnology, Nantong, China). Protein concentration was determined by the Bradford method. Protein was separated using sodium dodecyl sulfate-polyacrylamide gel electrophoresis (SDS-PAGE) and electroblotted onto a polyvinylidene fluoride (PVDF) membrane (Pall, Ann Arbor) by standard procedures. Transferred blots were incubated sequentially with antibodies, including primary rabbit anti-TM (1 : 200), primary mouse anti-*β*-actin (1 : 3000), and HRP-conjugated secondary antibodies (1 : 3000). Protein bands were visualized with an enhanced chemiluminescence detection kit and recorded on radiographic film (FluorChem FC2, Alpha Innotech). The resulting images were analyzed with ChemiImager 4000 to determine the integrated density value of each protein band.

### 2.14. Statistical Analysis

Data were analyzed using Prism 4 (GraphPad Software, San Diego, CA, USA) and expressed as mean ± standard deviation (SD). Multiple groups were compared using one-way analysis of variance followed by the Tukey-Kramer test for post hoc comparisons. Statistical significance was set at *P* < 0.05.

## 3. Results

### 3.1. The Effect of bFGF on the Apoptosis of HUVEC Induced by Radiation

**Figure 1:**
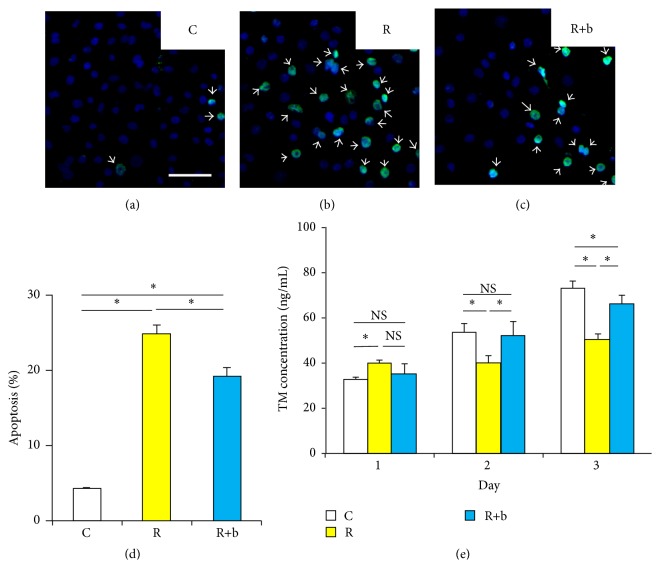
The effect of bFGF on apoptosis and TM expression of HUVEC exposed to radiation. (a)–(c) Representative images of TUNEL staining of each experimental group. White bar indicates 100 *μ*m. (d) Percentage of Annexin-V/PI double stained apoptotic cells determined by using flow cytometry. (e) The effects of bFGF on the TM expression in the medium of radiation treated HUVEC at different time points. NS, no significance, ^*∗*^ *P* < 0.05, NS: no significance. Bars represent mean ± SD from 3 independent experiments.

TUNEL staining was used to determine apoptotic cells in HUVEC. As shown in Figures 1(a)–1(c), the number of apoptotic cells increased after radiation. Pretreatment with bFGF showed protective effect on decreasing the apoptosis induced by radiation. To further quantify the apoptosis induced by radiation, Annexin-V/PI double stained cells were analyzed by flow cytometry. As shown in Figure 1(d) and Figure  S.2, the percentage of apoptotic cells reduced significantly in the R+b group compared with R group.

### 3.2. The Effect of bFGF on the TM Expression of HUVEC Exposed to Radiation

The concentration of TM in medium of HUVEC was applied to evaluate endothelial dysfunction. TM expression in control group increased day after day due to the proliferation of HUVECs. TM expression in radiated HUVEC decreased significantly at 2 and 3 days after radiation (Figure 1(e)). bFGF increased TM expression in HUVEC 2 and 3 days after exposure to radiation. However, bFGF did not increase the TM expression in radiated HUVEC 24 hours after radiation.

### 3.3. The Effect of bFGF on Urothelium Barrier at the Early Phase of RIBI

**Figure 2:**
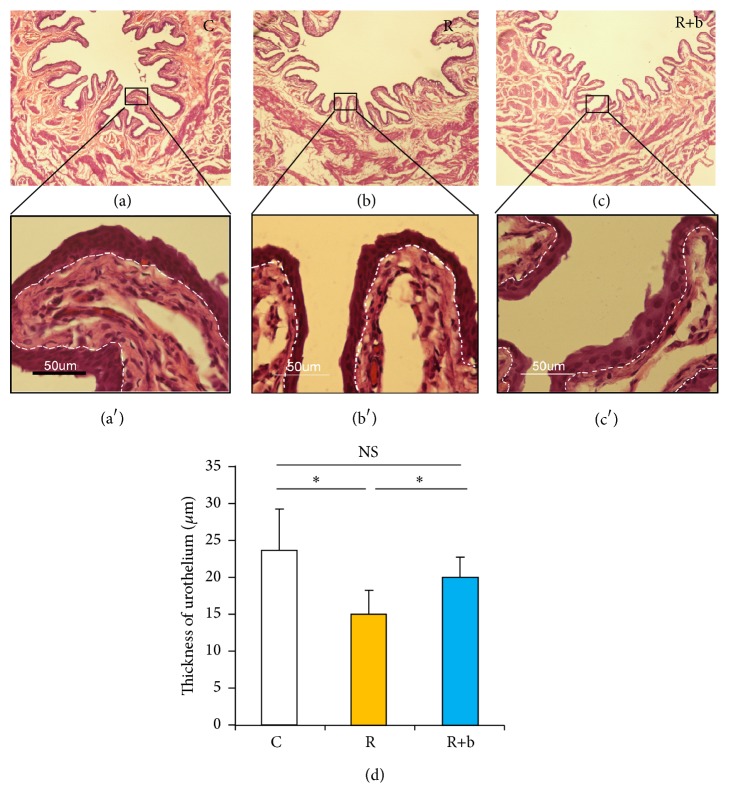
The effect of bFGF on urothelium at the early phase of RIBI. (a)–(c) Representative images of HE stained urinary bladder sections. Magnification is 40x. High magnifications in the boxed area further show the thickness of urothelium in each group ((a)′–(c)′). (d) Results of the thickness of the urothelium from each experimental group. ^*∗*^ *P* < 0.05, NS: no significance.

The thickness of urothelium was measured to reflect the urothelium barrier function [18]. 1 week after exposure to radiation, the thickness of urothelium decreased significantly in the R group compared with the C group (Figure 2). The thickness of urothelium in the R+b group increased significantly compared with the R group. bFGF did not increase the thickness of urothelium at the delayed phase of RIBI (Figure  S.5).

### 3.4. The Effect of bFGF on Inflammation in Urinary Bladder at the Early Phase of RIBI

**Figure 3:**
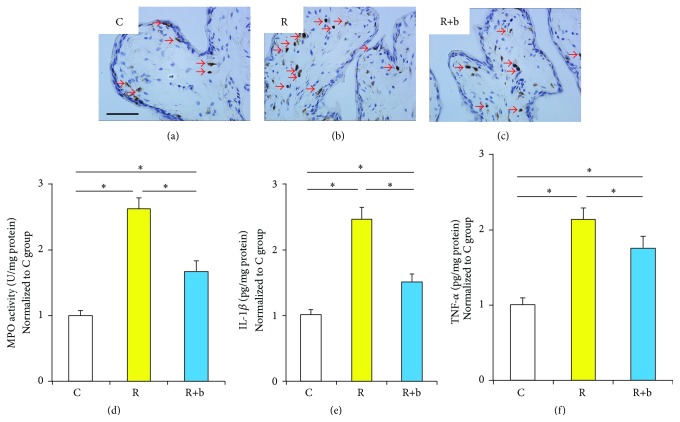
The effect of bFGF on inflammation in urinary bladder at the early phase of RIBI. (a)–(c) Representative images of bladder tissue immunohistologically stained with MPO. Black bar indicates 50 *μ*m. (d) Results of the MPO activity in bladder tissue. ^*∗*^ *P* < 0.01 compared with the R group. (e)-(f) Expressions of IL-1*β* (e) and TNF-*α* (f) in bladder tissue indicated by ELISA. ^*∗*^ *P* < 0.05.

Infiltration of MPO positive neutrophils and the expressions of inflammatory cytokines including TNF-*α* and IL-1*β* were used to evaluate inflammation in urinary bladder after radiation. The number of infiltrated neutrophils in group C is limited. Increased neutrophils were observed in the submucosa of group R compared with group C. The number of MPO positive cells decreased significantly in group R+b compared with group R (Figures 3(a)–3(c)). MPO activity in bladder tissue was measured to further quantify MPO positive neutrophils. As shown in Figure 3(d), the MPO activity reduced significantly in the R+b group compared with R group. The expressions of IL-1*β* and TNF-*α* increased significantly in the R group. The level of IL-1*β* and TNF-*α* in the bFGF treated group reduced significantly compared with group R (Figures 3(e) and 3(f)). Significance of acute inflammatory changes in urinary bladder was not observed between each experimental group at the delayed phase of RIBI (data was not shown).

### 3.5. The Effect of bFGF on Bladder Function at the Delayed Phase of RIBI

Urinary frequency and urinary volume per void investigated by metabolic cage were used to evaluate urinary bladder function. As shown in Figure  S.4, urinary bladder dysfunction after exposure to radiation was indicated by significantly increased urinary frequency and decreased urinary volume per void during the 24-hour period. Partial but significant recovery of bladder function was observed in group R+b. This is reflected by significantly decreased urinary frequency and increased urine volume. Significance of fibrotic changes in urinary bladder was not observed between each experimental group at the early phase of RIBI (data was not shown).

### 3.6. The Effect of bFGF on Bladder Fibrosis at the Delayed Phase of RIBI

**Figure 4:**
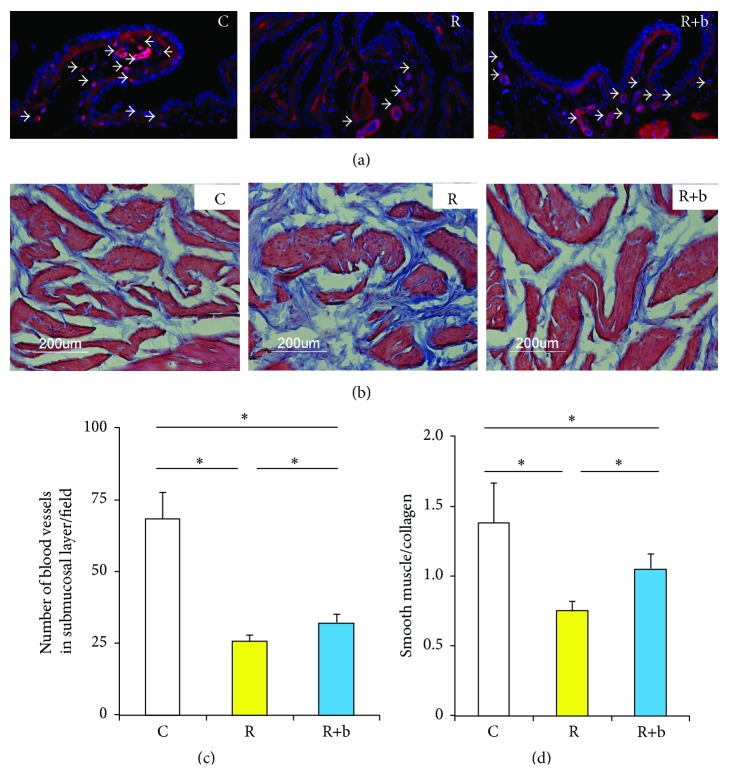
The effect of bFGF on bladder fibrosis at the delayed phase of RIBI. (a) Representative images of blood vessels in the submucosa of each experimental group. White bar indicates 100 *μ*m. (b) Representative images of Masson's trichrome staining of each experimental group. Results of the number of blood vessels in the submucosa (c) and the ratio between smooth muscle and collagen (d) in each group. ^*∗*^ *P* < 0.05.

The number of blood vessels in the submucosa area and the ratio between smooth muscle and collagen in the smooth muscle layer were used to evaluate bladder fibrosis. The results indicated that radiation could induce the vascular damage in the submucosa area, which was reflected by a significantly reduced number of blood vessels. Administration of bFGF significantly repaired the vascular damage, which was reflected by an increased number of blood vessels (Figures 4(a) and 4(c)). Fibrosis was observed in group R, reflected by a significantly reduced ratio of smooth muscle to collagen (Figures 4(b) and 4(d)). Administration of bFGF significantly increased the ratio of smooth muscle to collagen.

### 3.7. The Effects of bFGF on TM Expression in Urinary Bladder Exposed to Radiation

**Figure 5:**
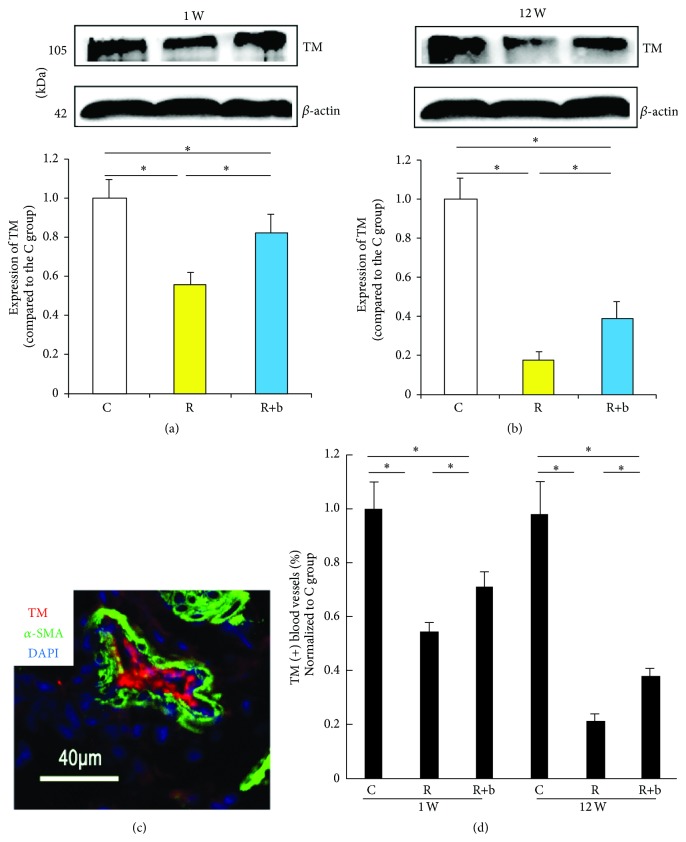
The effect of bFGF on TM expression in urinary bladder treated with radiation. (a)-(b) TM expression in urinary bladder 1 week (a) and 12 weeks (b) after exposure to radiation in each experimental group. ^*∗*^ *P* < 0.01 compared with the R group; ^#^ *P* < 0.05 compared with the R group. (c) Representative image of TM and *α*-SMA double stained urinary bladder section. (d) Results of the percentage of TM positive blood vessels within the urinary bladder. ^*∗*^ *P* < 0.05.

The Western blot data revealed that 1 week after exposure to radiation, the expression of TM in the bladder tissue decreased significantly in group R compared with group C (Figure 5(a)). bFGF preserved endothelial dysfunction at the early phase of RIBI, which was reflected by increased expression of TM in group R+b. Similarly, the TM expression in bladder tissue decreased significantly in group R compared with group C 12 weeks after radiation. bFGF was effective in preserving TM expression at the delayed phase of RIBI, which was indicated by the significantly increased TM expression in group R+b compared to group R (Figure 5(b)). The histological data revealed that the number of TM positive vessels increased significantly in group R+b 1 week and 12 weeks after exposure to radiation compared with group R (Figures 5(c) and 5(d)).

## 4. Discussion

Endothelial cells participate in multiple physiological functions, including maintenance of blood fluidity, control of vasomotor tone, trafficking of cells and nutrients, and maintenance of antithrombotic and anticoagulant balance [19]. An increasing body of evidence indicates that radiation-induced endothelial injury plays a central role in early and delayed radiation-induced injures in a variety of normal tissues [20]. In the early 21st century, Kolesnich demonstrated that endothelial apoptosis appeared to contribute heavily to early intestinal radiation toxicity [13]. Although there has been considerable controversy related to the extent and significance of endothelial apoptosis in the development of radiation-induced intestinal injury, numerous studies have shown that radiation-induced gastrointestinal syndrome is associated with endothelial cell injury [21, 22] and therapeutic strategies targeting endothelial protection have been indicated to be effective in alleviating the negatives effects of radiation on mice [22, 23]. Some scholars proposed that endothelial apoptosis may be just one of the manifestations of the dysfunctional state of endothelial cells. Therefore, endothelial dysfunction could be considered a main pathological change in the development of radiation-induced intestinal injury [6].

Loss of thromboresistance is a major feature of endothelial dysfunction after exposure to radiation [6]. TM is a particularly important marker reflecting radiation-induced endothelial dysfunction. TM can form a complex with thrombin and essentially converts thrombin from a procoagulant to an anticoagulant by changing its substrate specificity. When combined with TM, thrombin activates protein C, thereby limiting further thrombin generation and counteracting the inflammatory and fibroproliferative effects of thrombin [6]. Clinical and preclinical data has demonstrated that radiation caused a striking decrease of TM expression in vitro [15] and in vivo [16]. Furthermore, therapeutic strategies addressing TM preservation or thrombin inhibition are effective in minimizing side effects due to radiation exposure [24, 25]. Therefore, TM has been considered an important marker to reflect radiation-induced endothelial dysfunction.

Pathological changes of blood vessels have been reported in urinary bladders treated with radiation in mice [7–9], suggesting the potential role of endothelial dysfunction in the pathogenesis of RIBI. From our results, TM expression in the urinary bladder significantly reduced 1 week and 12 weeks after exposure to radiation (Figure 5), which confirms that endothelial dysfunction occurs in the early and delayed phases of RIBI. It is reasonable to speculate that endothelial dysfunction is one of the main features of radiation-induced pathological impairment in urinary bladder.

As an endothelial protective factor, bFGF has been demonstrated to be effective in inhibiting radiation-induced endothelial apoptosis in vitro [26] and in vivo [13]. The results from our study indicate that bFGF also has protective effects on HUVEC (Figure 1) against radiation-induced endothelial apoptosis, which was consistent with previously reported results [13]. Furthermore, our results also demonstrate that bFGF treatment partially prevents endothelial dysfunction, reflected by significant improved expression of TM in bFGF treated HUVEC or bladder (Figures 1 and 4). This data suggests that bFGF could be an ideal agent to rescue radiation-induced endothelial dysfunction.

The impairment of the urothelium barrier and the resulting leakage of urine are the generally accepted pathophysiological changes at the early phase of RIBI [18]. However, the mechanisms involved in radiation-induced impairment to urothelium are not fully understood. Many studies have proven that DNA damage is the main mechanism involved in radiation-induced damage [1, 3]. DNA damage commonly leads to death of cells in the first cell division or within the first few divisions [1]. Therefore, tissues with high proliferation rates, such as the epithelium of the intestine or the oral mucosa, are sensitive to radiation. Symptoms develop when functional cells are lost as part of normal tissue turnover and are not replaced because of damage to the stem cell compartment [1, 27]. In contrast with intestinal, the turnover time of the urothelium in rodents is reported to be much longer, in the range of 42–206 days [28]. However, the number of superficial cells in urothelium starts to decrease and the clinical symptoms appear early after radiation treatment [18]. This suggests that some other mechanisms besides radiation-induced direct impairment to urothelium may be involved in radiation-induced pathogenetic changes at the early phase of RIBI. Based on our findings in the present study, endothelial dysfunction may play a crucial role at the early phase of RIBI. Radiation-induced endothelial dysfunction might adversely affect the supply of oxygen and nutrients to that area. Also, abundant inflammatory cells may infiltrate into the bladder due to the increased permeability of microvasculature. From our in vivo data, intramuscular delivery of bFGF was shown to be effective not only in restoring damaged urothelium (Figure 2), but also in reducing inflammation within the bladder wall (Figure 3). Considering the crucial role of endothelial dysfunction and the effects of bFGF in rescuing endothelial dysfunction, it is reasonable to speculate that the bFGF might restore urothelium damage and counteract inflammation partly through rescuing endothelial dysfunction. In addition, it has been demonstrated that bFGF has protective effects on the proliferation, migration, and wound healing of urothelium [29], which might be another mechanism involved in bFGF ameliorating radiation-induced urothelial injury.

Deposition of collagen and fibrosis is the prominent feature of radiated urinary bladder at the delayed phase. However, the mechanisms involved in the fibrogenic process at the delayed phase of RIBI are not yet known. It has been well described that radiation-induced deficiency of TM is the key factor for radiation-induced intestinal fibrosis [16]. Radiation can cause TM deficiency in the endothelial cells, leading to insufficient scavenging of locally formed thrombin, which exerts proinflammatory and profibrogenic effects on mesenchymal cells. In the present study, a significant decrease in expression of TM was observed in the bladder tissue treated with radiation at the delayed phase (Figure 5), suggesting the possible association between endothelial dysfunction and bladder fibrosis.

Even bFGF was delivered immediately after radiation, the positive effects of bFGF could still be observed at the delayed phase, indicated by preserved bladder function (Figure  S.4) and promoted histology (Figure 4). It has been indicated that the risk for the development of delayed symptoms of RIBI is significantly increased as a consequence of the early reaction to radiation [30, 31]. Therefore, pretreatment targeting prevention of endothelial dysfunction at the early phase of RIBI is effective in preserving bladder function and preventing bladder fibrosis at the delayed phase of RIBI.

Our study has some limitations. First, bFGF was injected immediately after radiation, which has potential effect in promoting cancer growth if applied in clinical practice. Therefore, it would be interesting to investigate the effect of later treatment in preventing RIBI. Second, only one dosage of bFGF was injected in the present study. Further study aimed at investigating the dosage response of bFGF is needed.

In conclusion, our results indicate that endothelial dysfunction is a prominent feature in the pathogenesis of RIBI. bFGF was effective in preserving bladder function and histology partly by ameliorating endothelial dysfunction.

## Supplementary Material

Figure S1: Toxic effects of X-Ray on HUVECs. A. Different dose of X-Ray was delivered to HUVECs. Three days post radiation, measurement of cell viability was determined by CCK-8. ∗ P<0.01 compared with the control group, # P<0.05 compared with the control group. Bars represent mean ± SD from 6 independent samples. B. HUVECs exposed to various dosage of X-Ray stained with FDA/PI. FDA (green) indicates live cells while PI (red) indicates dead cells. The dose of 10Gy of X-ray was selected because it could induce significant decrease of cell viability without excessive death.
Figure S2: The effect of bFGF on the apoptosis of HUVEC induced by radiation. Representative images of Annexin-V/PI staining double stained apoptotic cells analyzed by using flow cytometry.
Figure S3: The effects of X-Ray on histology of urinary bladder. Different dose of X-Ray was radiated to the urinary bladder area of rats. Twelve weeks post radiation, rats were sacrificed and urinary bladder samples were collected for HE staining. A. Representative images of HE stained bladder tissues. B. Results of the number of blood vessels in the submucosa layer. ∗ P<0.01 compared with the C group. The dosage of 20Gy for animal study was selected because significant histological changes (reduced number of blood vessels in the submucosa layer) in the bladder wall were observed in rats radiated with 20Gy without excessive death of rats.
Figure S4: The effect of bFGF on bladder function at the delayed phase of RIBI.A. Representative images of metabolic cage data from each group. B-C. Results of urinary frequency (b) and urine volume per void (c) from each group. ∗ P<0.01 compared with the R group, # P<0.05 compared with the R group.
Figure S5: The effect of bFGF on the thickness of urothelium at the delayed phase of RIBI. A-C. Representative images of HE stained urinary bladder sections. D. Results of the thickness of the urothelium from each experimental group. ∗: P<0.05, NS: no significance.
