# Supplementary material for: Basic Fibroblast Growth Factor Ameliorates Endothelial Dysfunction in Radiation-Induced Bladder Injury
Source: Biomed Res Int. 2015 Aug 13;2015:967680. doi: 10.1155/2015/967680 (PMC4550748; doi:10.1155/2015/967680)
Supplement: Supplementary file 1 — Figure S1: Toxic effects of X-Ray on HUVECs. A. Different dose of X-Ray was delivered to HUVECs. Three days post radiation, measurement of cell viability was determined by CCK-8. ∗ P<0.01 compared with the control group, # P<0.05 compared with the control group. Bars represent mean ± SD from 6 independent samples. B. HUVECs exposed to various dosage of X-Ray stained with FDA/PI. FDA (green) indicates live cells while PI (red) indicates dead cells. The dose of 10Gy of X-ray was selected because it could induce significant decrease of cell viability without excessive death. Figure S2: The effect of bFGF on the apoptosis of HUVEC induced by radiation. Representative images of Annexin-V/PI staining double stained apoptotic cells analyzed by using flow cytometry. Figure S3: The effects of X-Ray on histology of urinary bladder. Different dose of X-Ray was radiated to the urinary bladder area of rats. Twelve weeks post radiation, rats were sacrificed and urinary bladder samples were collected for HE staining. A. Representative images of HE stained bladder tissues. B. Results of the number of blood vessels in the submucosa layer. ∗ P<0.01 compared with the C group. The dosage of 20Gy for animal study was selected because significant histological changes (reduced number of blood vessels in the submucosa layer) in the bladder wall were observed in rats radiated with 20Gy without excessive death of rats. Figure S4: The effect of bFGF on bladder function at the delayed phase of RIBI.A. Representative images of metabolic cage data from each group. B-C. Results of urinary frequency (b) and urine volume per void (c) from each group. ∗ P<0.01 compared with the R group, # P<0.05 compared with the R group. Figure S5: The effect of bFGF on the thickness of urothelium at the delayed phase of RIBI. A-C. Representative images of HE stained urinary bladder sections. D. Results of the thickness of the urothelium from each experimental group. ∗: P<0.05, NS: no significance. [file 967680.f1.docx]

**Supplementary Material**

**
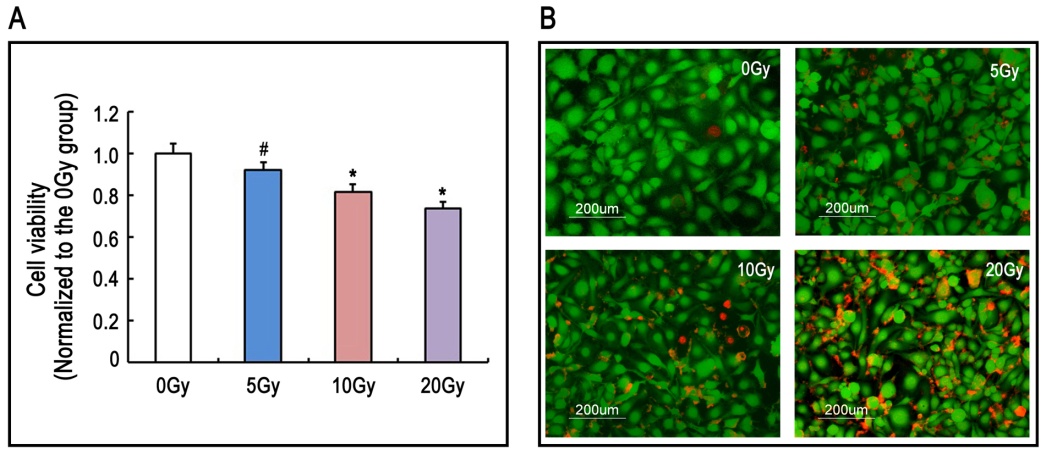
**

***Figure.S.1.*** *Toxic effects of X-Ray on HUVECs.* A. Different dose of X-Ray was delivered to HUVECs. Three days post radiation, measurement of cell viability was determined by CCK-8. * P<0.01 compared with the control group, # P<0.05 compared with the control group. Bars represent mean ± SD from 6 independent samples. B. HUVECs exposed to various dosage of X-Ray stained with FDA/PI. FDA (green) indicates live cells while PI (red) indicates dead cells. The dose of 10Gy of X-ray was selected because it could induce significant decrease of cell viability without excessive death.


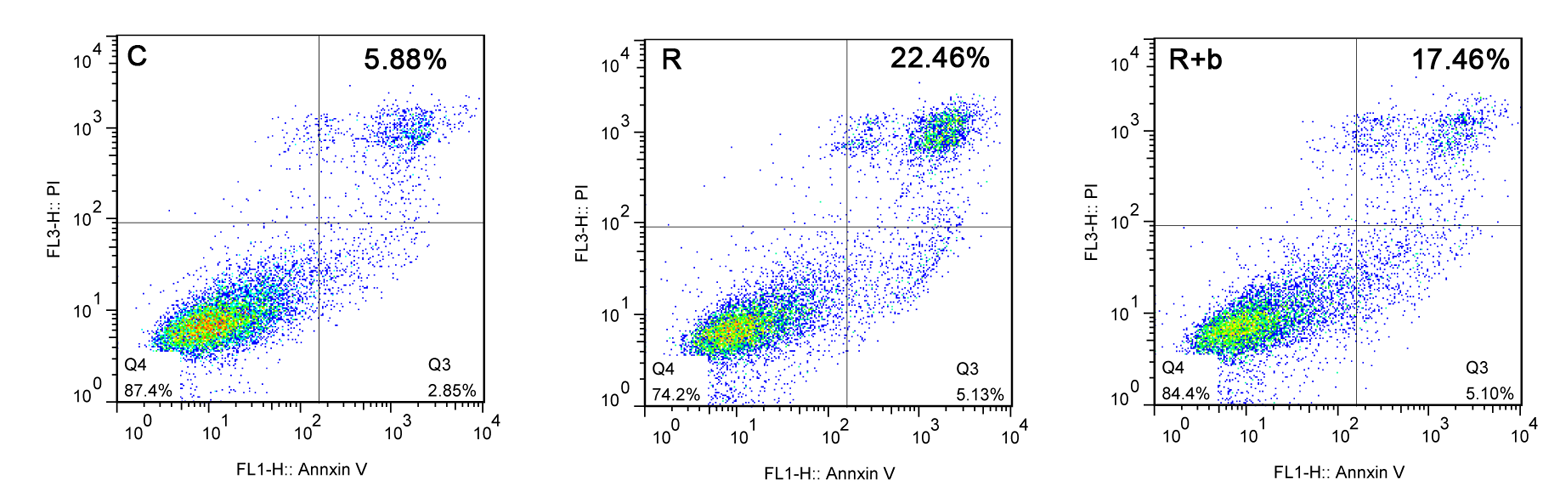


***Figure.S.2.*** *The effect of bFGF on the apoptosis of HUVEC induced by radiation.* Representative images of Annexin-V/PI staining double stained apoptotic cells analyzed by using flow cytometry.


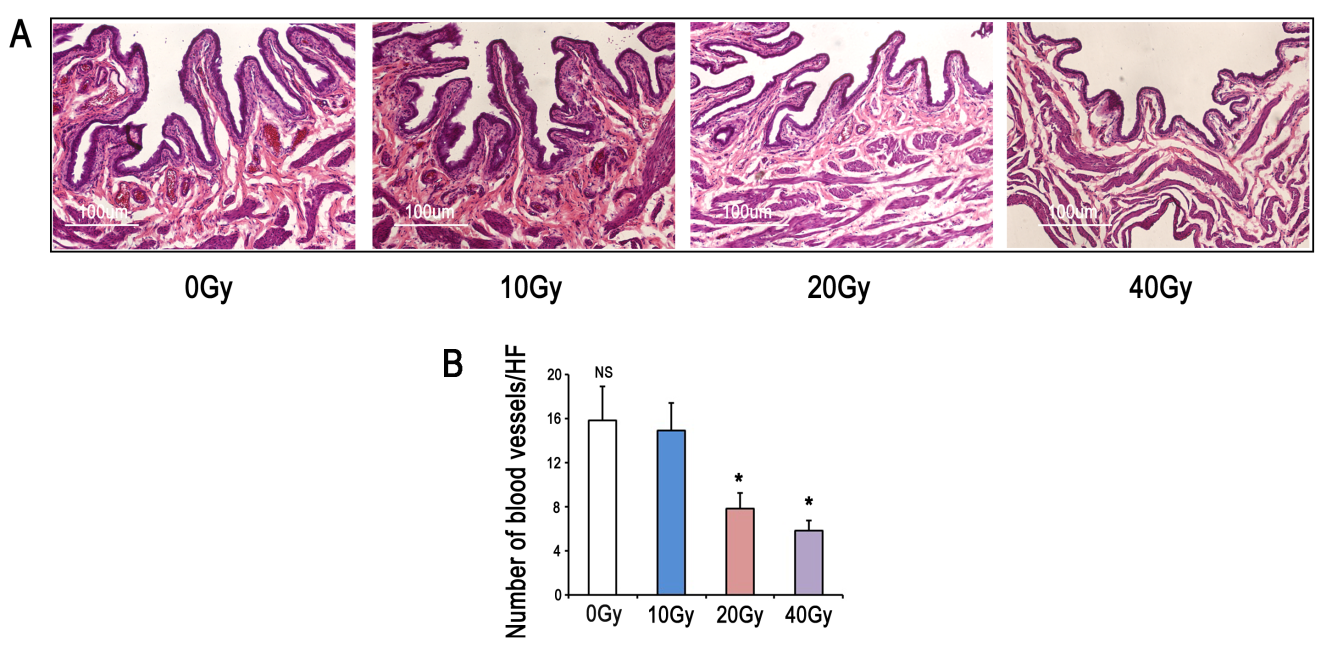


***Figure.S.3.*** *The effects of X-Ray on histology of urinary bladder.* Different dose of X-Ray was radiated to the urinary bladder area of rats. Twelve weeks post radiation, rats were sacrificed and urinary bladder samples were collected for HE staining. A. Representative images of HE stained bladder tissues. B. Results of the number of blood vessels in the submucosa layer. * P<0.01 compared with the C group. The dosage of 20Gy for animal study was selected because significant histological changes (reduced number of blood vessels in the submucosa layer) in the bladder wall were observed in rats radiated with 20Gy without excessive death of rats.


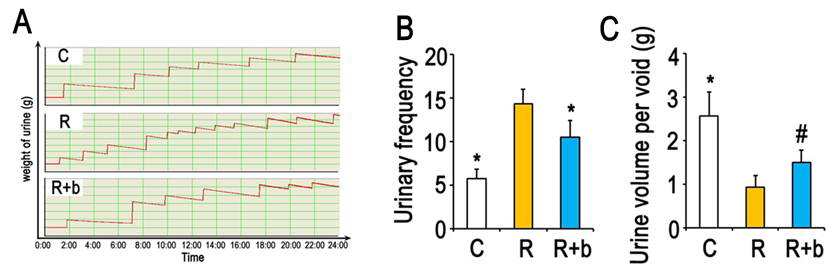


***Figure.S.4*** *The effect of bFGF on bladder function at the delayed phase of RIBI.*A. Representative images of metabolic cage data from each group. B-C. Results of urinary frequency (b) and urine volume per void (c) from each group. * P<0.01 compared with the R group, # P<0.05 compared with the R group.


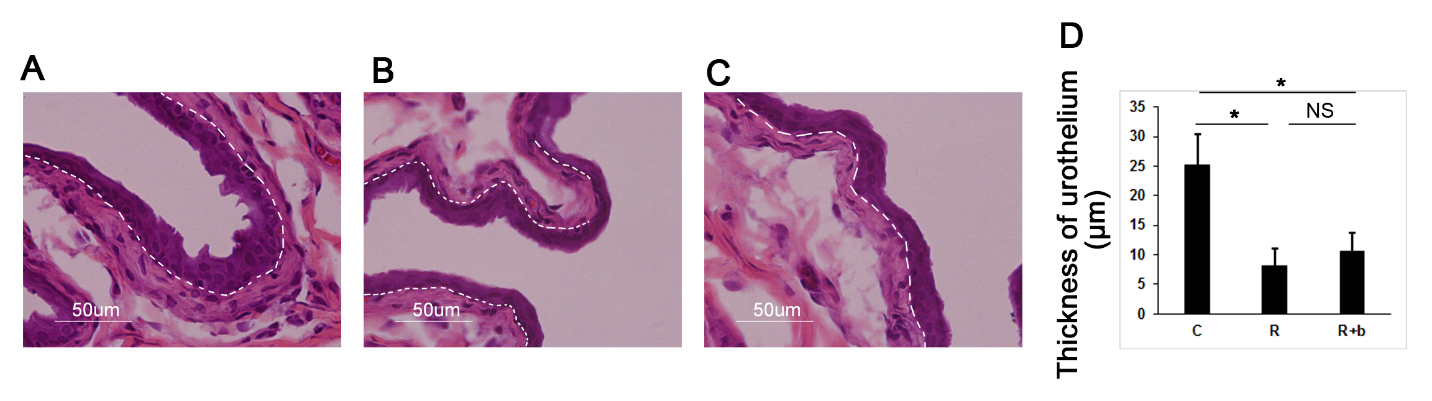


***Figure.S.5*** *The effect of bFGF on the thickness of urothelium at the delayed phase of RIBI.* A-C. Representative images of HE stained urinary bladder sections. D. Results of the thickness of the urothelium from each experimental group. *: P<0.05, NS: no significance.
